# Health-Related Quality of Life and Risk Factors among Chinese Women in Japan Following the COVID-19 Outbreak †

**DOI:** 10.3390/ijerph18168745

**Published:** 2021-08-19

**Authors:** Yunjie Luo, Yoko Sato

**Affiliations:** 1 Graduate School of Health Sciences, Hokkaido University, Hokkaido 060-0812, Japan; raa@eis.hokudai.ac.jp; 2 Faculty of Health Sciences, Hokkaido University, Sapporo, Hokkaido 060-0812, Japan

**Keywords:** COVID-19, health, Chinese women, immigrant women, immigrants, quality of life, mental health, physical health, SF-36v2

## Abstract

The COVID-19 pandemic has significantly affected individuals’ physical and mental health, including that of immigrant women. This study aimed to evaluate the health-related quality of life (HRQoL), identify the demographic factors and awareness of the COVID-19 pandemic contributing to physical and mental health, and examine the risk factors associated with poor physical and mental health of Chinese women in Japan following the COVID-19 pandemic outbreak. Using an electronic questionnaire survey, we collected data including items on HRQoL, awareness of the COVID-19 pandemic, and demographic factors. One hundred and ninety-three participants were analyzed. Approximately 98.9% of them thought that COVID-19 affected their daily lives, and 97.4% had COVID-19 concerns. Married status (OR = 2.88, 95%CI [1.07, 7.72], *p* = 0.036), high concerns (OR = 3.99, 95%CI [1.46, 10.94], *p* = 0.007), and no concerns (OR = 8.75, 95%CI [1.17, 65.52], *p* = 0.035) about the COVID-19 pandemic were significantly associated with poor physical health. Unmarried status (OR = 2.83, 95%CI [1.20, 6.70], *p* = 0.018) and high COVID-19 concerns (OR = 2.17, 95%CI [1.04, 4.56], *p* = 0.040) were significantly associated with poor mental health. It is necessary to provide effective social support for Chinese women in Japan to improve their well-being, especially in terms of mental health.

## 1. Introduction

Since December 2019, the novel coronavirus SARS-CoV-2 has been spreading rapidly throughout the world. As of 25 June 2021, more than 179 million coronavirus disease-2019 (COVID-19) cases have been confirmed, while more than 3.8 million people have succumbed to the disease [1]. In Japan, the first COVID-19 case was reported on 16 January 2020 [2]. As of March 2021, Japan has reported four waves of increasing COVID-19 cases [3]. Moreover, judging from the latest data in May 2021, Japan seems to have reached the fifth peak of increasing COVID-19 cases [4]. As of 27 June 2021, the number of infected individuals in Japan exceeded 794,000, while the number of deaths has reached 14,657 [5].

Numerous countries and regions have taken various prevention and control measures and strategies to reduce the spread of COVID-19 transmission, such as lockdowns, imposing physical distancing, staying at home, and restricting group events [6,7]. In Japan, the government urged residents to establish a “new lifestyle” based on avoiding places corresponding to the “three Cs” (closed spaces, crowded places, and close-contact settings) and implementing necessary countermeasures such as physical distancing, wearing a mask, and washing hands [8]. Although these measures have proven effective in slowing the spread of the disease, they inevitably resulted in a substantial negative effect on mental and physical health and well-being among various populations [9,10,11].

An existing study reported that some of the COVID-19 prevention measures had significantly affected the mental health of people living in Japan [12]. Due to the COVID-19 outbreak, the general female population in Japan experienced poor mental health, with a significantly increased suicide rate [13]. Immigrant women, as a minority group, experienced more mental health problems than native women [14]. A qualitative study on the immigrant community in the United States (U.S.) reported that the COVID-19 pandemic affects immigrants’ mental health and interpersonal relationships [15]. We inferred that immigrant women in Japan might experience a low level of mental health status due to the outbreak of the COVID-19 pandemic.

The COVID-19 lockdown reduced the general population’s physical activity, which harmed their physical health [16,17]. Due to the COVID-19 restrictions, the resultant long daily sitting hours and unhealthy eating behaviors were found to have a negative effect on public physical health and activity [18]. On the other hand, the stay-at-home policy and perceived time availability were positively related to healthy food literacy, which could improve individuals’ physical health [19]. A study reported that the general Chinese population experienced poor physical health, and women had lower physical activity than men did during the COVID-19 pandemic [20]. However, the physical health status of immigrant women in Japan during the current pandemic has not yet been clarified.

Chinese women comprise the largest population of foreign women in Japan, accounting for 29.4% of all foreign women as of June 2020 [21]. Chinese immigrant women encounter difficulties due to vulnerabilities associated with their gender responsibilities and the acculturation process, which may influence their physical and mental health [22]. A finding reported that Chinese women in Japan undergo acculturation stress and are at a higher risk for mental disorders [23]. Due to the ongoing pandemic, the Chinese government declared a lockdown from 23 January 2020, and announced stricter testing and two-week quarantine measures for people wanting to enter China [24]. These measures placed the Chinese women in Japan in a stressful situation and made it challenging for them to return to China. Moreover, an earlier study reported that more than half of the Chinese citizens experienced mental health problems as a common response to the pandemic; these included anxiety, depression, and self-reported stress [25]. Therefore, we proposed that the health status of Chinese women in Japan might have been severely affected by the COVID-19 outbreak, thus necessitating further investigation.

Health-related quality of life (HRQoL) reflects an individual’s perceived well-being in physical and mental domains of health [26]. To date, many studies have been conducted examining the HRQoL of various populations to demonstrate their physical and mental health status, before and after the COVID-19 pandemic outbreak [20,27,28,29]. A previous study evaluated the HRQoL of Chinese immigrant women in Japan and found that they experienced a low level of mental health before the COVID-19 outbreak [30]. Hence, it is necessary to investigate the HRQoL of Chinese women in Japan, which will not only help to evaluate their health status but also could determine the potential risk factors for their poor physical and mental health in the ongoing COVID-19 period.

A part of this study was presented at the 3rd International Electronic Conference on Environmental Research and Public Health [31]. This study had the following three objectives.

- Objective 1. To assess the HRQoL scores of Chinese women in Japan following the COVID-19 pandemic outbreak;
- Objective 2. To identify the demographic characteristics and awareness of the COVID-19 pandemic contributing to physical and mental health;
- Objective 3. To examine the risk factors associated with poor physical and mental health among Chinese women in Japan following the COVID-19 pandemic outbreak.

## 2. Materials and Methods

### 2.1. Data Collection and Participants

This study was performed following the COVID-19 pandemic outbreak, from 1 to 31 October 2020. Due to the COVID-19 prevention control measures, group events in community settings for foreigners in Japan were suspended. The survey was difficult to conduct using a paper-pen questionnaire, which is usually distributed through community settings such as an international communication plaza. Therefore, an online anonymous questionnaire survey was created for Chinese women in Japan using Qualtrics Survey Software, a web-based survey tool developed by an American experience management company called Qualtrics. Participants were recruited from various online groups of Chinese residents in Japan using WeChat, a Chinese multi-purpose messaging social media platform developed by Tencent. The snowball sampling method was used to recruit more participants. The inclusion criteria were as follows: (1) aged older than 20 years; (2) Chinese women living in Japan following the COVID-19 pandemic outbreak; (3) able to speak, read, and understand Mandarin Chinese.

### 2.2. Measures

#### 2.2.1. Health-Related Quality of Life

HRQoL was evaluated using the 36-Item Short-Form Health Survey, version 2.0 (SF-36v2), which was developed as part of the Medical Outcomes Study. SF-36v2 is a general health survey for evaluating the mental and physical health status of various populations [32,33]. A simplified Chinese version of the SF-36v2 was used; its reliability and validity have been previously tested [28,34,35]. The SF-36v2 consists of 36 items on eight subscales and measures two dimensions. The eight subscales include physical functioning (PF), role-physical (RP), bodily pain (BP), general health (GH), vitality (VT), social functioning (SF), role-emotional (RE), and mental health (MH). The two dimensions are the mental component summary (MCS) and the physical component summary (PCS). PRO CoRE software provided by QualityMetric, Inc., was used to calculate the scores. The scores range from 0 to 100, with a higher score indicating a higher level in that health subscale. Norm-based t-scores were used to calculate the scores of each subscale and dimension, with a mean of 50 and a standard deviation of 10. Scores below 47 indicate a risk of impaired functioning in the associated subscale and domain [36]. All of the subscales and domains of HRQoL in this study sample demonstrated a substantial or almost perfect Cronbach’s alpha coefficient (0.696 to 0.905).

#### 2.2.2. Participants’ Awareness of the COVID-19 Pandemic

Two items with defined response choices were designed to understand the participants’ awareness of the pandemic. Item 1 was “The degree of the effect of the COVID-19 pandemic on your daily life,” with “high,” “low,” and “none” as the response options. Item 2 was “The degree of your concern regarding the COVID-19 pandemic,” with “high,” “low,” and “none” as the response choices.

#### 2.2.3. Demographic Characteristics

Based on the literature [22,23,30,37], brief demographic characteristics were collected, including age, marital status, duration of residence in Japan, planned length of stay, educational background, status of residence, and annual household income.

### 2.3. Data Analysis

All data analyses were conducted using IBM SPSS Statistics version 26.0 (IBM, New York, NY, USA). The categorical variables were analyzed using the descriptive statistics method. The Shapiro–Wilk normality test was used to evaluate the distribution of continuous variables. For normally distributed data, the mean and standard deviation (SD) of continuous variables were calculated, and comparisons of the mean scores of the covariate groups were undertaken using the Student’s *t*-test and one-way analysis of variance. For non-normally distributed data, the median and interquartile range (IQR) of continuous variables were calculated, and the Mann–Whitney U test and the Kruskal–Wallis test were used to examine the significant differences between the covariate groups. The Spearman’s rank correlation test for continuous data was conducted to evaluate their association with HRQoL scores.

To evaluate the potential risk factors of poor physical and mental health, univariate analysis and multivariable logistic regression analysis were performed. Univariate analysis was conducted as a pre-selection test for all variables. Since a relaxed *p*-value criterion could reduce the risk of missing important predictor variables [38,39], we included the variables with a *p*-value less than 0.1 in univariate analysis in the multivariate model. Odds ratios (OR) and 95% confidence intervals (CI) from the logistic regression analysis were presented to show the association between the predictor variables and poor physical and mental health. The Hosmer–Lemeshow test was performed to assess the model’s goodness-of-fit [40]. The Cronbach’s alpha of each HRQoL subscale and domain was calculated. Results with a *p*-value of less than 0.05 were considered significant.

### 2.4. Ethics Statement

The study was approved by the ethical review committee of the Faculty of Health Sciences, Hokkaido University, Japan (approval no. 20–28). Each participant provided informed consent before completing the questionnaire. All data will be retained by us for five years.

## 3. Results

### 3.1. Response Rate and Descriptive Characteristics of Participants

A total of 203 women responded to and submitted the questionnaires. Among the 203 responses, 10 (4.9%) did not complete the SF-36v2 scale and were thus excluded. Finally, 193 (95.1%) participants were included and analyzed for Objectives 1 and 2. The descriptive characteristics of the 193 participants are shown in Table 1. Thirty-four participants did not answer data for age, marital status, duration of residence in Japan, planned length of stay, status of residence, and annual household income. Thus, these missing data were excluded (n = 34), and the valid data (n = 159) were included to analyze the potential risk factors of poor physical and mental health for Objective 3.

The mean age of the study sample was 27.88 (±5.84) years, ranging from 20 to 51 years old. The majority of participants were unmarried (79.3%), residing in Japan for less than three years (57.0%), had a planned length of stay in Japan of less than three years (57.0%), had completed graduate school or above (64.2%), had non-work permit status of residence (68.4%), and were earning less than JPY 3,000,000 annually (64.2%). In our study sample, approximately 98.9% of the participants thought that the COVID-19 pandemic affected their daily lives, and 55.4% thought that the effect was high. Approximately 97.4% of the participants had COVID-19 concerns, while 51.8% felt that their concerns were reduced.

### 3.2. HRQoL Scores among Chinese Women in Japan Following the COVID-19 Pandemic Outbreak

**Figure 1:**
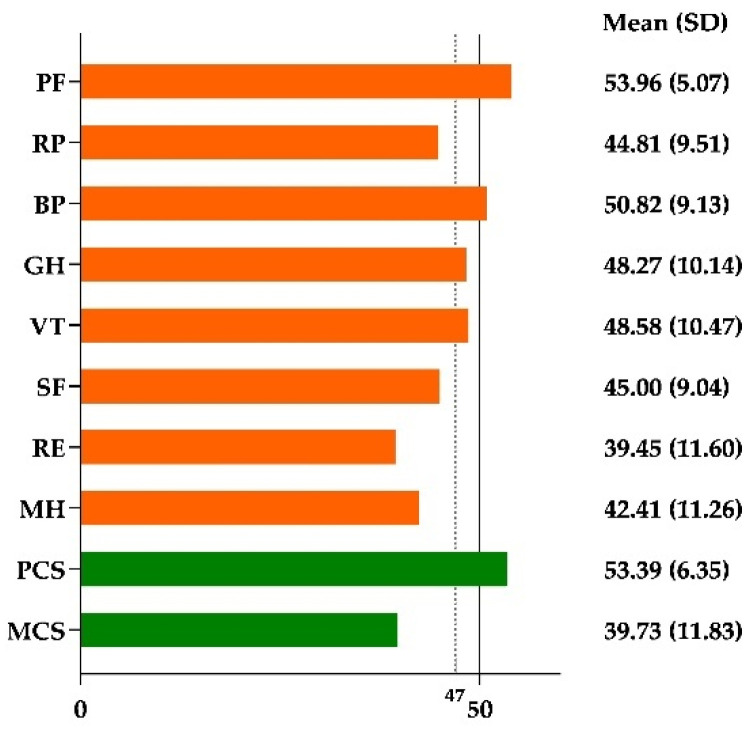
Mean scores of each SF-36v2 subscale and domain following the COVID-19 pandemic outbreak (n = 193). (PF: physical functioning; RP: role-physical; BP: bodily pain; GH: general health; VT: vitality; SF: social functioning; RE: role-emotional; MH: mental health; PCS: physical component summary; MCS: mental component summary; SD: standard deviation).

Figure 1 shows the mean scores of the SF-36v2 subscales and dimensions. Chinese women in Japan had lower scores for RP, GH, VT, SF, RE, MH, and MCS than the mean scores of 50 in the general population. Additionally, the mean scores of RP, SF, RE, MH, and MCS were lower than 47, which indicated a risk of impaired functioning in these health subscales and domains.

### 3.3. Participants’ Descriptive Characteristics Contributing to Physical and Mental Health

The descriptive characteristics of participants contributing to physical and mental health are presented in Table 2. Unmarried women showed a significantly higher physical health status than married women (*p* = 0.013). Women who felt that the pandemic had highly affected their life had a significantly low mental health status (*p* = 0.037), whereas women with high concerns regarding the pandemic showed a significantly lower mental status than women with low or no concerns (*p* = 0.000).

### 3.4. Risk Factors Associated with Poor Physical and Mental Health of Chinese Immigrant Women Following the COVID-19 Pandemic Outbreak

To perform the logistic regression analysis, participants were stratified into two groups according to their PCS and MCS scores, with a cutoff point of 47.

Table 3 shows the results of the univariate analysis and multivariate logistic regression analysis of the risk factors for poor physical health. In the multivariable model, the results demonstrated that being married (OR = 2.88, 95%CI [1.07, 7.72], *p* = 0.036) and having high concerns (OR = 3.99, 95%CI [1.46, 10.94], *p* = 0.007) and no concerns (OR = 8.75, 95%CI [1.17, 65.52], *p* = 0.035) about the COVID-19 pandemic were significant factors associated with poor physical health. The *p*-values of the Hosmer–Lemeshow goodness-of-fit test for poor physical models were 0.093.

Table 4 shows the results of the univariate analysis and multivariate logistic regression analysis of the risk factors for poor mental health. Being unmarried (OR = 2.83, 95%CI [1.20, 6.70], *p* = 0.018) and having high concerns about the COVID-19 pandemic (OR = 2.17, 95%CI [1.04, 4.56], *p* = 0.040) were significantly associated with poor mental health. The *p*-values of the Hosmer–Lemeshow goodness-of-fit test for poor mental health models were 0.989.

## 4. Discussion

To the best of our knowledge, this study is the first to examine the HRQoL of Chinese women living in Japan following the COVID-19 pandemic outbreak. These women experienced a low level of mental health and a standard level of physical health. This study provides evidence for policymakers, public health nurses, and social support providers to improve the physical and mental health and well-being of immigrant women during a crisis period such as a pandemic.

Following the COVID-19 pandemic outbreak, the majority of detained Chinese women in Japan were young, unmarried newcomers with high educational backgrounds, non-work permit visas, and low household incomes. This study’s relatively young, detained immigrant population was similar to that in an earlier finding involving detained immigrants in the U.S. [41]. In another study, over half of the Chinese women in Japan thought that the effect of COVID-19 on their daily lives was high, and over 40% of them felt that their COVID-19 concerns were high. The rate of Chinese women in Japan who felt stressed about the COVID-19 pandemic is higher than that of the general Chinese adult population [20] and Chinese stroke patients during the COVID-19 pandemic [27].

Compared to the norms for Japanese women [42], the mean scores of all mental health-related subscales and domains (VT, SF, RE, MH, and MCS) and partial physical health-related subscales (RP and GH) were low among Chinese women in Japan following the COVID-19 pandemic outbreak. The results share several similarities with an existing study of Chinese immigrant women in Japan before the COVID-19 outbreak [31]. These poor health results, whether before or after the COVID-19 pandemic, reveal that the Chinese women living in Japan generally experience health issues, especially that of well-being in the mental health domain. An existing study reported that during the COVID-19 pandemic, the residence status of immigrants is a significant risk factor for poor health among immigrant women [43]. Many foreign workers in Japan have lost their jobs due to the pandemic [44], which may have further negatively influenced their health status. Considering our findings, it is clear that the well-being of Chinese women in Japan needs to be addressed and that social support to improve their physical and mental health status is necessary, especially in the ongoing pandemic.

Chinese women in Japan who were highly affected by the COVID-19 pandemic had a considerably low mental health status. The effect of COVID-19 on mental health has also been addressed in many studies involving various populations [9,45,46]. One possible reason is the lifestyle changes required to manage the spread of COVID-19. Although preventive measures, such as staying at home and restrictions on events, had the intended temporary effect on controlling the spread of COVID-19, people’s socioeconomic activities were significantly affected, leading to increased stress and anxiety [47,48]. Women, in particular, have been more severely affected in terms of mental health issues, compared to men [49]. With the changes in lifestyle, women are likely to experience a greater psychological effect and develop avoidant behaviors when facing the challenges of the COVID-19 pandemic [50]. Another possible explanation is that Chinese women in Japan were limited due to their immigrant status, with economic difficulties and language barriers, which are associated with a low mental health level. A finding among Brazilian immigrants reported a similar psychological burden [15].

Marital status was found to have a significant effect on women’s physical and mental health [51]. The results indicated a considerable risk of impaired physical health among married Chinese women in Japan following the COVID-19 pandemic outbreak. One reason for married women suffering from physical health impairment may be that they are usually the primary caregivers of their families [52], and the pandemic resulted in increasing their work burden, while also taking care of their families at home [53]. However, the unmarried status of Chinese women in Japan is a risk factor for impaired mental health functioning. Unlike married Chinese women in Japan who live with their families, unmarried Chinese women in Japan usually live alone. During the COVID-19 pandemic, the stay-at-home orders meant that those who live alone and potentially socially isolated were at a higher risk of developing loneliness and depression than people living with someone else [54]. Loneliness and depression are specific risk factors for mental health problems, especially in the period of the COVID-19 pandemic [55,56].

COVID-19 concerns were significantly associated with mental health issues among various populations, such as pregnant women and general adolescents [57,58]. Our findings identified that Chinese women in Japan with high concerns about the COVID-19 pandemic showed not only an increased risk of poor mental health but also poor physical health. This finding is similar to a previous study, which reported that concerns about the COVID-19 pandemic are significantly associated with the physical and mental health of the general Chinese adult population [20]. In addition, we found an interesting result that the absence of COVID-19 concerns is significantly associated with poor physical health. Unlike people with COVID-19 concerns who have taken coping strategies such as exercising and eating healthy food [59], Chinese women in Japan with no COVID-19 concerns might lack the necessary health-related coping strategies to maintain or improve their physical health.

Due to the COVID-19 pandemic outbreak, all support and events to assist the adaption process of foreign residents, such as interpretation services, childcare seminars, and child-rearing meet-ups, have been suspended. Other studies indicated that due to the COVID-19 restrictions, various populations experienced a low level of social activity, which consequently influenced their physical and mental health [60,61]. The lack of necessary social activities may be a noteworthy reason for the effect on immigrant Chinese women’s physical and mental health. We suggest that the policymakers need to update the support plan for immigrant women to provide available social support—such as online seminars and meet-ups—during these extraordinary periods to improve their health status.

Of course, several limitations may have influenced the results of this study. First, selection bias may have occurred. The study samples did not cover all regions of Japan, which may have affected the generalizability of this study’s findings. Additionally, the data were collected online, which could be the reason why the study sample was young. The study’s cross-sectional design is also a limitation. A follow-up study using a qualitative research design is suggested to further explore the health-related experience of immigrant women in Japan. A previous study indicated that to elucidate the influence of the COVID-19 pandemic on people’s physical and mental health, surveys at two points—before and after the peak of COVID-19—are critical [62]. However, there is a lack of health data on general Chinese women living in Japan before the COVID-19 outbreak. Further investigations using longitudinal studies to explore the risk factors of physical and mental health examined in this study should be considered.

## 5. Conclusions

Following the COVID-19 pandemic outbreak, over 95% of Chinese women in Japan reported that the pandemic affected their daily lives and resulted in concerns about the pandemic. Chinese women in Japan experienced a low level of mental health well-being. Marital status significantly contributed to the physical health of Chinese women in Japan. Meanwhile, COVID-19 effects and concerns significantly contributed to their mental health. The multivariate logistic regression model showed that married status, high COVID-19 concerns, and absence of COVID-19 concerns were associated with poor physical health. Moreover, unmarried status and high COVID-19 were associated with poor mental health. This study provides evidence for policymakers, public health nurses, and social supporters to maintain and improve the well-being of Chinese immigrant women and immigrant women in Japan, especially in terms of mental health, during the ongoing pandemic. It is necessary to provide effective social support for Chinese women in Japan to improve their mental health status, such as developing an evidence-based mental health improvement program.

## Figures and Tables

**Table 1:** Descriptive characteristics of participants (n = 193).

Variables	Number	%
Age (years)		
20–29	139	72.0
30–39	38	19.7
40–49	4	2.1
≥50	4	2.1
No answer	8	4.1
Marital status		
Married	39	20.2
Unmarried	153	79.3
No answer	1	0.5
Duration of residence in Japan (years)		
<3	110	57.0
≥3	82	42.5
No answer	1	0.5
Planned length of stay (years)		
<3	110	57.0
≥3	71	36.8
No answer	12	6.2
Educational background		
Primary school	1	0.5
Middle school	3	1.6
High school	3	1.6
College	3	1.6
University	59	30.6
Graduate school or above	124	64.2
Status of residence		
Work permit	58	30.1
Non-work permit	132	68.4
No answer	3	1.6
Annual household income (JPY)		
<3,000,000	124	64.2
≥3,000,000	56	29.0
No answer	13	6.7
The degree of the effect of the COVID-19 pandemic on participants’ daily lives		
High	107	55.4
Low	84	43.5
None	2	1.0
The degree of participants’ concern about the COVID-19 pandemic		
High	88	45.6
Low	100	51.8
None	5	2.6

JPY: Japanese Yen.

**Table 2:** Participants’ descriptive characteristics contributing to PCS and MCS scores (n = 193).

Variables	PCS		MCS	
	Mean (SD)	*p*-Value	Median (IQR)	*p*-Value
Age (years)	0.028 ^1^	0.730 ^2^	−0.003 ^1^	0.970 ^2^
Marital status				
Married	51.19 (6.76)	0.013 ^3^	46.33 (18.08)	0.078 ^4^
Unmarried	54.00 (6.11)		40.48 (14.18)	
Duration of residence in Japan (years)				
<3	53.18 (6.10)	0.586 ^3^	40.79 (13.94)	0.803 ^4^
≥3	53.68 (6.72)		40.72 (16.57)	
Planned length of stay (years)				
<3	53.47 (5.83)	0.535 ^3^	40.51 (15.28)	0.310 ^4^
≥3	52.87 (7.15)		41.67 (15.28)	
Educational background				
High school or below	48.80 (7.68)	0.135 ^3^	30.11 (26.46)	0.853 ^4^
University or college	53.26 (7.32)		41.54 (13.74)	
Graduate school or above	53.71 (5.67)		40.18 (15.47)	
Status of residence				
Work permit	53.74 (6.56)	0.621 ^3^	41.72 (16.28)	0.288 ^4^
Non-work permit	53.24 (6.34)		40.18 (14.22)	
Annual household income (JPY)				
<3,000,000	53.01 (6.03)	0.417 ^3^	39.89 (13.45)	0.339 ^4^
≥3,000,000	53.85 (7.18)		41.95 (17.52)	
The degree of the effect of the COVID-19 pandemic on participants’ daily lives				
High	52.98 (6.60)	0.090 ^5^	39.45 (16.07)	0.037 ^4^
Low	53.68 (5.93)		41.38 (15.50)	
None	62.57 (0.02)		49.55	
The degree of participants’ concern about the COVID-19 pandemic				
High	52.61 (7.46)	0.359 ^5^	36.25 (12.98) ^6^	0.000 ^5^
Low	54.07 (5.04)		42.30 (9.76) ^6^	
None	53.25 (8.24)		49.54 (12.05) ^6^	

^1^ Spearman’s rank correlation coefficients; ^2^ Spearman’s rank correlation test; ^3^ Student’s *t*-test; ^4^ Mann–Whitney U test; ^5^ One-way analysis of variance; ^6^ Mean (standard deviation); PCS: physical component summary; MCS: mental component summary; IQR: interquartile range; SD: standard deviations; JPY: Japanese Yen.

**Table 3:** Univariate analysis and multivariate logistic regression analysis of risk factors for poor physical health with PCS scores less than 47 (n = 159).

Variables	Poor Physical Health (PCS Scores < 47)			
	Univariate Analysis		Multivariable Analysis	
	OR [95% CI]	*p*-Value	OR [95% CI]	*p*-Value
Age (years)	1.03 [0.96, 1.11]	0.424		
Marital status				
Unmarried (Ref)				
Married	3.21 [1.25, 8.25]	0.016	2.88 [1.07, 7.72]	0.036
Duration of residence in Japan (years)				
≥3 (Ref)				
<3	1.89 [0.80, 4.46]	0.149		
Planned length of stay (years)				
≥3 (Ref)				
<3	2.01 [0.85, 4.76]	0.114		
Educational background				
High school or below (Ref)				
University or college	0.36 [0.05, 2.43]	0.293		
Graduate school or above	0.22 [0.03, 1.44]	0.114		
Status of residence				
Work permit (Ref)				
Non-work permit	0.57 [0.24, 1.39]	0.216		
Annual household income (JPY)				
<3,000,000 (Ref)				
≥3,000,000	1.03 [0.41, 2.58]	0.948		
The degree of the effect of the COVID-19 pandemic on participants’ daily lives				
Low or none (Ref)				
High	1.33 [0.56, 3.17]	0.519		
The degree of participants’ concern about the COVID-19 pandemic				
Low (Ref)				
High	4.30 [1.59, 11.64]	0.004	3.99 [1.46, 10.94]	0.007
None	8.78 [1.22, 63.09]	0.031	8.75 [1.17, 65.52]	0.035

PCS: physical component summary; JPY: Japanese Yen.

**Table 4:** Univariate analysis and multivariate logistic regression analysis of risk factors for poor mental health with MCS scores less than 47 (n = 159).

Variables	Poor Mental Health (MCS Scores < 47)			
	Univariate Analysis		Multivariable Analysis	
	OR [95% CI]	*p*-Value	OR [95% CI]	*p*-Value
Age (years)	0.99 [0.93, 1.05]	0.675		
Marital status				
Married (Ref)				
Unmarried	2.44 [1.07, 5.55]	0.034	2.83 [1.20, 6.70]	0.018
Duration of residence in Japan (years)				
≥3 (Ref)				
<3	0.83 [0.42, 1.62]	0.577		
Planned length of stay (years)				
≥3 (Ref)				
<3	0.60 [0.31, 1.19]	0.143		
Educational background				
High school or below (Ref)				
University or college	3.09 [0.47, 20.25]	0.240		
Graduate school or above	3.60 [0.57, 22.65]	0.172		
Status of residence				
Work permit (Ref)				
Non-work permit	1.77 [0.87, 3.62]	0.116		
Annual household income (JPY)				
<3,000,000 (Ref)				
≥3,000,000	0.57, [0.28, 1.15]	0.118		
The degree of the effect of the COVID-19 pandemic on participants’ daily lives				
Low or none (Ref)				
High	1.43 [0.73, 2.80]	0.298		
The degree of participants’ concern about the COVID-19 pandemic				
Low (Ref)				
High	1.90 [0.93, 3.88]	0.077	2.17 [1.04, 4.56]	0.040
None	0.38 [0.06, 2.42]	0.307	0.39 [0.06, 2.56]	0.329

MCS: mental component summary; JPY: Japanese Yen.

## Data Availability

Not applicable.
